# The Association Between Social Determinants of Health and Population Health Outcomes: Ecological Analysis

**DOI:** 10.2196/44070

**Published:** 2023-03-29

**Authors:** Ace Vo, Youyou Tao, Yan Li, Abdulaziz Albarrak

**Affiliations:** 1 Information Systems and Business Analytics Department Loyola Marymount University Los Angeles, CA United States; 2 Center for Information Systems and Technology Claremont Graduate University Claremont, CA United States; 3 Information Systems Department King Faisal University Al-Ahsa Saudi Arabia

**Keywords:** social determinants of health, public policy, health outcomes, policy recommendation, cities

## Abstract

**Background:**

With the increased availability of data, a growing number of studies have been conducted to address the impact of social determinants of health (SDOH) factors on population health outcomes. However, such an impact is either examined at the county level or the state level in the United States. The results of analysis at lower administrative levels would be useful for local policy makers to make informed health policy decisions.

**Objective:**

This study aimed to investigate the ecological association between SDOH factors and population health outcomes at the census tract level and the city level. The findings of this study can be applied to support local policy makers in efforts to improve population health, enhance the quality of care, and reduce health inequity.

**Methods:**

This ecological analysis was conducted based on 29,126 census tracts in 499 cities across all 50 states in the United States. These cities were grouped into 5 categories based on their population density and political affiliation. Feature selection was applied to reduce the number of SDOH variables from 148 to 9. A linear mixed-effects model was then applied to account for the fixed effect and random effects of SDOH variables at both the census tract level and the city level.

**Results:**

The finding reveals that all 9 selected SDOH variables had a statistically significant impact on population health outcomes for ≥2 city groups classified by population density and political affiliation; however, the magnitude of the impact varied among the different groups. The results also show that 4 SDOH risk factors, namely, asthma, kidney disease, smoking, and food stamps, significantly affect population health outcomes in all groups (*P*<.01 or *P*<.001). The group differences in health outcomes for the 4 factors were further assessed using a predictive margin analysis.

**Conclusions:**

The analysis reveals that population density and political affiliation are effective delineations for separating how the SDOH affects health outcomes. In addition, different SDOH risk factors have varied effects on health outcomes among different city groups but similar effects within city groups. Our study has 2 policy implications. First, cities in different groups should prioritize different resources for SDOH risk mitigation to maximize health outcomes. Second, cities in the same group can share knowledge and enable more effective SDOH-enabled policy transfers for population health.

## Introduction

### Overview

Social determinants of health (SDOH), defined by the World Health Organization, encompass economic policies, social and physical environments, and access to health services and shapes the conditions “in which people are born, grow, work, live, and age” [1].

It is well established that SDOH factors, such as health behaviors, clinical care, social and economic status, and physical environment, account for 30% to 55% of health outcomes [2]. There is considerable literature examining the association between SDOH factors and various health outcomes, among which most studies used patient-level and hospital-level data. For example, prior studies have found that SDOH factors are associated with medication adherence [3], care use [4], readmission risk [5,6], length of stay [7,8], postoperative surgical outcomes [9], mortality risk [10,11], and risk of exposure to and subsequent health outcomes after contracting the SARS-CoV-2 virus [12,13].

Understanding the ecological association between SDOH factors and population-level health outcomes is vital. Such an understanding is particularly relevant for researchers, clinicians, and policy makers in assessing new SDOH-enabled programs or policies to improve population health, enhance quality of care, and reduce health inequities [14,15]. Several frameworks have been developed to understand the impact of SDOH risk factors on population health outcomes [16-19]. Among these frameworks, the County Health Ranking (CHR) model has been widely applied to explicate the relationship between SDOH factors and population health outcomes in the United States [17,20].

The CHR model uses >30 ranking criteria to measure the impact of SDOH factors on the current and future population health outcomes at the county level [17]. However, this model is not always accurate at the state level or lower-than-state level (eg, county, city, and census tract level) because it uses a predetermined set of weights for the SDOH factors. Two recent empirical studies of the CHR found that the influence of SDOH factors on health outcomes varies among different states in the United States [21,22]. In addition, existing CHR studies are limited to either the county level or the state level. Analysis at a lower level is desired by local policy makers because local governments need to derive insights into the SDOH and health outcomes within their administrative delineation [23]. For instance, Corburn et al [23] showed substantial results in reducing health inequities by examining all policies adopted by the city of Richmond, California, at the zip code level.

This study investigates the ecological associations between the SDOH and population health outcomes at the city level to support health policy decisions using the CHR as the foundation. For this purpose, we curated data from 5 different sources and integrated them at the census tract level from 29,126 census tracts within 499 cities across all 50 states in the United States. With such smaller geographical delineations, researchers, policy makers, and other relevant parties can aggregate data into larger administrative divisions to make more impactful and effective decisions.

After grouping cities through 2 factors, namely, population density and political affiliation, we formalized measures for SDOH factors and population health outcomes and used a voting-based feature selection approach to reduce the original 148 sociodemographic and SDOH-related variables to 9. We then used a linear mixed-effects model to examine the ecological associations between the SDOH factors and population health outcomes. At the census tract level, the goodness of fit ranges from 0.65 to 0.75. At the city level, the total variance explained by the model was high, ranging from 0.86 to 0.90. The effect size of variables is different across groups. Noticeably, asthma, kidney disease, smoking, and food stamp variables were significant in all groups. Post hoc analysis was later conducted using predictive margin to assess group differences in health outcomes for the 4 behavioral health indicators that majorly affect health outcomes across all groups.

### Background

#### Social Determinants of Health

SDOH encompass a wide set of dimensions such as socioeconomics, education, physical environment, food access, health care system condition, health behaviors, community status, and politics [1,24,25]. The importance of the SDOH is apparent to both academia and policy makers. Research on SDOH is impactful and prominent, especially in the context of this research, that is, linking health outcomes and disparities [2,3,12,26,27].

In the United States, the CHR model is a widely applied model that describes how SDOH factors contribute to population health [17,20]. The CHR model uses >30 ranking measures to understand the current population health outcomes and health factors (ie, health behaviors, clinical care, social and economic status, and physical environment) that would affect future health in the United States [17]. Both the health outcomes and health factors are weighted by a panel of national experts on population health in the CHR model [17].

Although the CHR model makes health outcomes and factors easy to calculate and be understood by the general public and policy makers, it has 2 major drawbacks. First, the weights proposed by experts in the CHR may not be applicable to the entire population because different locations may have various population characteristics and dissimilar social and policy environments. Two recent studies that empirically tested the CHR [21,22] revealed that the influence of health factors on health outcomes varies among the US states. In other words, the CHR model performed better in some states than in others. Second, previous studies used SDOH through CHR at either the county level or the state level. A more granular level of data is needed to comprehensively understand the SDOH factors that affect population health and to provide insights for local policy makers, such as governments at the city level. In this study, we use census tract data, a geographical delineation of a county that encompasses approximately 4000 population, with relatively well-maintained and updated demographic information [28]. Using census tracts as the unit of analysis, researchers and policy makers can aggregate the data into larger administrative divisions.

Generally, SDOH research has both breadth and depth. Research can be found using study sites worldwide, with different takes on what SDOH are, and using a diverse range of research methods. The same perspective can be gleamed when focusing on the relationship between health outcomes and the SDOH. Owing to the divergent and seemingly agglomeration research that explores the relationship between health outcomes and SDOH factors, researchers need to be careful when defining (1) the health outcomes measurement, (2) the SDOH factors, (3) the population and study sites, and (4) the research methods. These definitions set up the phenomenon for research while providing concrete findings to help promote population health, which in turn affects policy changes.

#### SDOH Factors and Health Outcomes

Health outcomes and SDOH factors are interlinked. For instance, poverty, education, and income are known to be closely related to health outcomes [12,26,29-32]. Specifically, Washington et al [30] identified low income to be related to the higher unmet needs for health care, whereas Bauer et al [33] found a major association between income and health literacy. Several studies have associated income and insurance status with risk of readmission [6,34]. Similarly, studies have found that income, age, and vulnerable populations have worse postoperative surgical outcomes or higher mortality risk [9-11]. Furthermore, when present alongside SDOH, disadvantaged racial and ethnic groups are negatively affected in several health outcomes areas, including COVID-19 [35], stroke [36], and kidney transplant [37].

Health behavior is another essential component of SDOH [38,39]. It is related to people’s health practices such as smoking, diet, exercise, and alcohol habits. According to Healthy People 2020, individuals’ healthy behaviors can substantially influence their health needs and outcomes [40]. Likewise, it was estimated that approximately 443,000 Americans die annually of smoking-related diseases, such as cancer, stroke, lung disease, and heart disease [41]. Lack of physical activities and high-calorie food intake can lead to obesity, which majorly affects people’s health conditions.

#### Population Density

At any location, the population can be delineated into living in 3 areas: urban, suburban, and rural. Although the general public could easily discern the differences between the 3, in research, this has been proven as not as clear-cut as it may seem [42]. Generally, there is a decision framework, along with several decision criteria, to designate a location to be either urban, suburban, or rural. This designation separates the way in which people live. Unfortunately, owing to the nature of our research inquiry that examines census tracts at the city level, the abovementioned delineation cannot be definitively justified. However, based on the simple criteria of population density, we were able to dissect cities into different segments, each of which had different health behaviors and outcomes.

Indeed, population density is closely related to health service delivery and further influences health outcomes. On the one hand, health care institutions located in low population–density places may need to manage small-scale operations and handle financial losses owing to low volume, whereas people who live in low population–density locations may have difficulties accessing health care facilities and services because of human service and resource deficiency. For example, in less populated areas, ambulance response time is likely to be longer [43].

Furthermore, these low population–density locations usually have an increasingly aging population, demanding more health services and resources [44,45]. Government-supported health programs are often limited in low population–density areas because of the high rate of poverty and limited tax in such areas [45], and high population–density areas may cause congestion in major hospitals [46], suggesting a complex relationship between population density and health outcomes. The interplay between the supply and demand of health care is greatly influenced by the availability of services and the needs of the population. Naturally, the denser the population, the more health care is needed. In contrast, low population–density areas require fewer health care services but suffer from scarcity.

Previous studies have found that population density is associated with various health outcomes, such as mortality rate [47,48], survival outcome [49], and morbidity in certain diseases [48]. To account for the complex relationship between population density and health outcomes, we examined the relationship between SDOH and health outcomes by grouping cities based on their population density.

#### Political Affiliation

Local governments and their political leaning greatly affect health policy. The US political system is a constant wrestle between 2 major political parties: the Democratic Party (often colorized as blue) and the Republican Party (often colorized as red). Contention exists throughout the United States and at various levels of the government. At the state level, the differences between the parties encompass both health policy and social issues (eg, attitude and policy regarding abortion and substance use) and the preferred role of the government (eg, big vs small government) in addressing health-related problems [50]. For example, Pagel et al [51] painted a stark contrast in the priorities of health care policy between Democratic and Republican state legislators. Republicans prioritize reducing health costs and smaller government, whereas Democrats prioritize improving health and equity and reducing disparities over other goals [51].

At the individual level, partisan polarization in public attitudes shapes individuals’ health behaviors. For example, studies found that there are diverging attitudes between Republicans and Democrats toward influenza and the COVID-19 vaccine, in which Republicans displayed a negative attitude and intention toward vaccine, whereas attitudes and intentions of Democrats remained largely stable [52,53]. Given the importance of how political affiliation can affect both policies and attitudes toward health outcomes, we surmise that the identification of political parties would also influence the relationship between health outcomes and SDOH.

Despite the research outcomes suggesting linkages between health outcomes and SDOH, policy makers do not view SDOH as a priority, as is evident in the absence of SDOH in the general government policy agenda, despite earlier emphasis [54,55]. Instead, current health policy in the United States still focuses on “medicalizing” health problems, assuming that the solution to health is medical care [55,56]. For example, policy makers often emphasized health access policies to increase geographic and financial access to health services for vulnerable populations [56] but neglected other important social and economic factors that are associated with health disparities [56]. Prior studies also pointed out that even with universal health care access, rich populations are more likely to have healthier lives because they can afford advanced health care; thus, the most important policy issues in health care are largely dependent on the overall allocation of resources to health care rather than merely on the distributive justice within health care [57,58].

Furthermore, Embrett and Randall [54] suggested that extant SDOH policy studies appear to be focused on advocacy rather than analysis. To promote “healthy public policies” based on SDOH factors, health disparities need to be examined so that the government can develop specific policy responses with their tools at hand such as regulation, legislation, taxation, and financing [59]. Health care professionals, researchers, and governments can work together to empirically examine the extent to which SDOH factors contribute to disparate health outcomes so that policies can be developed to solve health disparities in a more effective and efficient manner [13].

This study makes the following contributions to research and practice. First, the study divulges additional insights into how different SDOH factors affect health outcomes by curating related data in census tracts. In contrast to the county level, targeted but limited SDOH data are available at the census tract level. Our study presents a novel data curation process that creates additional SDOH variables that are otherwise not readily available. At the census tract level, data could be aggregated to the city level, allowing policy makers to devise policy more effectively. Second, city is a living and emergent ecosystem, and each city presents its own opportunities and challenges in terms of population health [60]. Despite these differences, our study sheds unique insights through 5 distinct groups with 2 prevailing properties: the city’s population density and its political affiliation. The analysis results showed that the health effectiveness intragroup was similar, whereas the effects diverged intergroup. Therefore, it is prudent for cities from different groups to prioritize resource allocations to address SDOH factors based on their group properties so that population health outcomes can be maximized.

## Methods

### Study Design

The study design includes 4 considerations. First, to perform the ecological analysis at the city level, we need to identify data sources for SDOH factors and health outcomes at the group level. Second, we need to determine the unit of analysis, that is, how to define the study population and the method of grouping. Third, we need to formalize the measures for SDOH factors and population health outcomes. Finally, we need to determine the appropriate data analysis method. In the subsequent sections, we describe each consideration in detail.

### Data Collection

We curated and integrated a data set from 5 different sources: the PLACES program [61], the National Center for Health Statistics, Census Data portal, Simply Analytics, and the Massachusetts Institute of Technology Election Laboratory [62]. The data obtained from the PLACES program included population health outcomes, such as mental and physical health days, as well as population health behaviors, such as the percentage of the population with asthma or kidney disease. Additional data were collected at the census tract level for a selected 500 cities in the United States, which serves as the basis of the data for analysis. Life expectancy data for each census tract were obtained from the National Center for Health Statistics [63]. These data were later used to measure the health outcomes. Population-related variables such as age, income, ethnicities, and education levels were obtained through the Census Data portal, supplemented by data from the Simply Analytics platform. We used data from the Massachusetts Institute of Technology Election Laboratory to determine the political affiliation to which each census tract belongs. Political affiliation is proxied by the 2020 presidential election. In the United States, voting for a presidential candidate happens in the general election every 4 years, in which most of the population votes, and it solidifies the general political direction of the country, whether it will lean toward Democratic or Republican policies. Election data are reported at the precinct level, which is a larger spatial delineation than census tracts, is smaller than US counties, and overlaps with US cities. When precincts have more votes for the Democrats presidential candidate, we code the census tracts to reside within it as blue. Similarly, we coded red for census tracts that reside in precincts that have more votes for the Republican presidential candidate. census tract data from Washington, District of Columbia, were dropped because they did not have a life expectancy measure. The final data set included 29,126 census tracts within 499 cities across all 50 states in the United States, all obtained in 2021.

### Determining Unit of Analysis

The results of our data collection process yielded the census tract as the default unit of analysis. We further examined whether this unit of analysis was sufficient or whether an additional grouping mechanism was warranted. As the census tracts resided within the city boundary, a natural grouping was to coalesce tracts based on the city itself. However, this type of grouping did not help explain the relationship between cities. Rather, additional grouping of cities was required. Therefore, we used political affiliation and population density, as discussed in the previous sections with the same name.

To determine the political affiliation of a city, we revisited the political affiliation of each census tract, which was determined using the abovementioned description. With a simple majority rule, if a city has more census tracts that are red, we assign red as the city’s political affiliation. Similarly, a city will be coded blue as its political affiliation if a majority of the census tracts residing within it are blue. Correspondingly, the population density of a tract was calculated by dividing the total population by its area in square miles [44,49]. Each city was then sorted and classified into 3 quartiles: sparsely populated cities were below the 25th percentile (denoted low), medium-populated cities were between the 25th and 75th percentiles (denoted mid), and highly populated cities were above the 75th percentile (denoted high). The final grouping included 73 cities in blue-low, 198 cities in blue-mid, 120 cities in blue-high, 53 cities in red-low, 51 cities in red-mid, and 4 cities in red-high. As red-high had a small number of cities, it was combined with the red-mid category, making the final number of cities in the combined category, red-high, 55. Multimedia Appendix 1 includes the list of cities in each classification.

### Formalizing Measurements

#### Formalizing Health Outcome Measurement

After integrating and cleaning the data, we then formulated the health outcomes measurements for our research based on the CHR model [17]. In the CHR model, health outcome measures the current state of population health and can be further divided into 2 categories: length of life and quality of life. Length of life can be measured by life expectancy and quality of life by poor physical health days and poor mental health days. Specifically, the health outcome measure was based on the CHR model, as shown in the following equation:


Health outcome = 50% × (life expectancy) − 25% × (poor physical health days) − 25% × (poor mental health days) (1)

Similar to the CHR model, our proposed health outcome measure assigns equal weightage to the length of life (through life expectancy) and quality of life (through poor physical health days and poor mental health days). The subtraction signs signify the negative effects of having poor physical health days and poor mental health days on health outcomes.

#### Formalizing SDOH Measurements

To formalize the measurements for SDOH factors, we first standardized the different scales such as rates, percentages, and averages of the survey responses. We also followed the CHR model by standardizing all measures with *z* scores, in which the standardized variables had a mean of 0 and a SD of 1 [17,22]. The initial SDOH data comprised 148 variables (Multimedia Appendix 2), including some highly correlated features. Thus, a feature reduction is required. As different feature selection techniques may result in a different set of features and no standardized rules on which technique might be better than the others, we experimented with the following 5 feature selection methods: Pearson correlation, recursive feature elimination, backward stepwise regression, XGBoost, and random forest. Independent variables were then ranked into 4 quartiles from highest to lowest, based on their feature importance or effect size against the dependent variable health outcome. We then applied a majority voting method, retaining variables that were placed in the top quartile based on at least 3 methods. The final data set included 9 variables for SDOH risk factors (Table 1).

**Table 1:** Metadata of the final data set^a^.

Variable name	Type	Description
Health outcome	DV^b^	Health outcome of the population (refer to equation 1)
Asthma	IV^c^	Percentage of the population aged >18 years and with asthma
Kidney disease	IV	Percentage of the population aged >18 years and with chronic kidney disease
Smoking	IV	Percentage of the population aged >18 years who has smoked >100 cigarettes in their lifetime and currently smoke
Teeth lost	IV	Percentage of the population aged ≥65 years who has lost all their natural teeth
Annual checkup	IV	Percentage of the population aged >18 years who has visited a physician for a routine checkup
Lack of sleep	IV	Percentage of the population aged >18 years who sleeps <7 hours over a 24-hour period
Lack of health insurance	IV	Percentage of the population aged between 18 and 64 years who does not have health insurance
Below poverty	IV	Percentage of the population below the federal poverty level
Food stamps	IV	Percentage of households using food stamps or other cash public assistance programs
Group	Grouping	Grouping of tracts based on the city’s population density and political affiliation (5 groups in total)

^a^The final data set included 1 dependent variable, 9 independent variables, and 1 grouping variable. Metadata applies for all 29,126 census tracts within 499 cities in the United States in 2021.

^b^DV: dependent variable.

^c^IV: independent variable.

### Data Analysis

For data analysis, a linear mixed-effects model [64] was used to analyze the statistical parameters that varied at the census tract level and the city level. Observations in census tracts were hypothesized to have a systematic and predictable influence on health outcomes. Thus, the census tract data were modeled as a fixed effect, nonrandom, or nonindependent. As different cities are expected to have unpredictable and nonsystematic effects, they were modeled as random effects.

### Ethical Considerations

The data used in this study are secondary data, which do not involve human participants, and are collected and aggregated by the respective organizations mentioned in the *Data Collection* section, most of which are curated from the US Census Bureau as the primary source. No additional data were collected specifically for this study.

## Results

All the linear mixed-effects models converged. We present the results of the linear mixed-effect models for 5 groups: blue-low (model 1), blue-mid (model 2), blue-high (model 3), red-low (model 4), and red-high (model 5) in Tables 2-4. The *R*^2^ results from the 5 models indicated that the overall fit of each model was adequate. Specifically, the conditional *R*^2^ represents the variance of the variables explained by both fixed and random effects within the model. The conditional *R*^2^ value ranging from 0.86 to 0.90 in models 1 to 5 indicated that the grouping at the city level exemplifies the intertwined relationship between population density and political affiliation grouping against health outcomes. Furthermore, the marginal *R*^2^ represents the variance affected by the fixed effects. The marginal *R*^2^ value ranging from 0.60 to 0.75 in models 1 to 5 indicates that the set of variables at the census tract level also has high goodness of fit to health outcomes.

Our results suggest that all the groups examined in the study have an equipotential baseline, as evidenced by the comparable intercepts across all 5 city groups classified by population density and political affiliation. Furthermore, the results showed that all SDOH variables had a statistically significant impact on population health outcomes for ≥2 or city groups, but the magnitude of the impact varied among the different groups. For example, an increase in the proportion of the population with asthma, kidney disease, smoking, or using cash assistance programs (such as food stamps) is associated with a decline in population health outcomes across all groups, consistent with the SDOH literature [65-68]. However, the magnitude of the impact of these variables on population health outcomes varied among different groups. The impact of asthma on population health outcomes is highest for cities classified as red-high (model 5: red-high) and lowest for cities classified as blue-low (model 1: blue-low), whereas the impact of kidney disease or smoking is higher for blue cities (as observed in models 1-3). The impact of food stamps on population health outcomes is higher for red cities with high population density (model 5: red-high) and blue cities with medium population density (model 2: blue-mid).

Some SDOH variables were statistically significant for a subset of the city groups. For instance, the percentage of the population having an annual checkup does not have a statistically significant impact on population health outcomes for red cities with low population density (model 4: red-low), whereas the impact of the percentage of the population below the federal poverty level is not statistically significant only for red cities with high population density (model 5: red-high). Similarly, the impact of the percentage of the population without health insurance is not statistically significant for blue cities with low density (model 1: blue-low). Finally, the impact of lack of sleep is only statistically significant for rural cities, and the impact of teeth loss is statistically significant for cities with a high population density (model 3: blue-high and model 5: red-high), regardless of their political affiliation.

**Figure 1:**
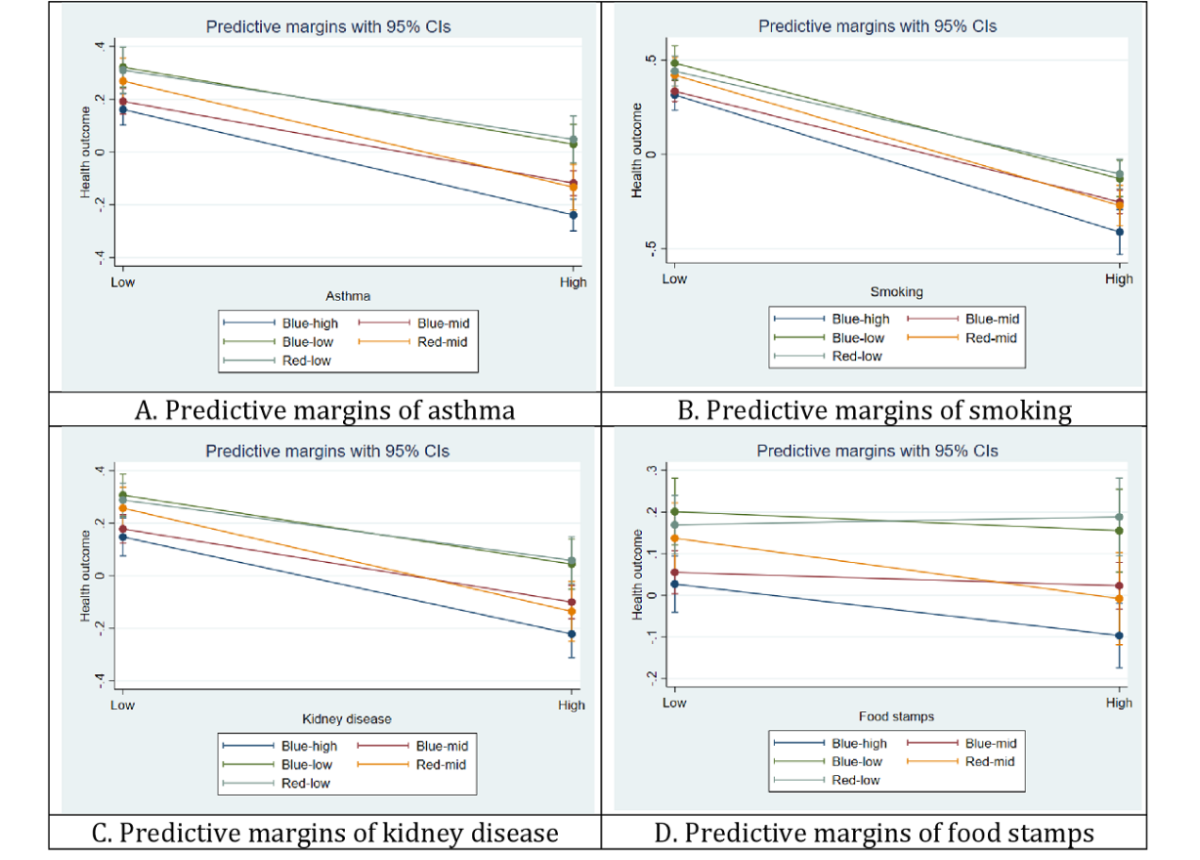
Post hoc analysis via predictive margins for (A) asthma, (B) smoking, (C) kidney disease, and (D) food stamps. Each colored line represents each city grouping. The x-axis shows whether the variable is high or low, where low means 1 SD below the mean and high means 1 SD above the mean. The y-axis displays the value of health outcome. This figure applies to all 29,126 census tracts within 499 cities in the United States in 2021.

To further investigate these findings, a post hoc analysis was conducted using predictive margins to assess group differences in health outcomes for the 4 SDOH variables, namely, asthma, kidney disease, smoking, and food stamps, that substantially affected health outcomes across all groups (as shown in Figure 1). Figure 1 illustrates that each group had a better health outcome with low asthma, low smoking, low kidney disease, or low food stamps and a worse health outcome with high asthma, high smoking, high kidney disease, or high food stamps. One exception was the red-low group, which had a slightly better health outcome with a high food stamp. Overall, the blue-low and red-low groups had better health outcomes than the other 3 groups, whereas blue-high had the lowest health outcome compared with the other 4 groups. Furthermore, asthma, kidney disease, smoking, and food stamps had larger impacts on the red-high and blue-high groups than the other 3 groups.

**Table 2:** Census tract level–fixed effect results of the linear mixed-effect model.

	Model 1 (blue-low)			Model 2 (blue-mid)			Model 3 (blue-high)			Model 4 (red-low)			Model 5 (red-high)	
	Coefficient (SE)	*P* value	Coefficient (SE)		*P* value	Coefficient (SE)		*P* value	Coefficient (SE)		*P* value	Coefficient (SE)		*P* value
Lack of health insurance	−0.124 (0.095)	.19	−0.284 (0.042)		<.001	−0.697 (0.038)		<.001	−0.792 (0.093)		<.001	−0.808 (0.138)		<.001
Asthma^a^	−0.848 (0.158)	<.001	−1.006 (0.109)		<.001	−1.248 (0.075)		<.001	−1.266 (0.228)		<.001	−1.731 (0.256)		<.001
Kidney disease^a^	−1.247 (0.130)	<.001	−1.491 (0.096)		<.001	−1.973 (0.092)		<.001	−0.738 (0.193)		<.001	−1.348 (0.284)		<.001
Smoking^a^	−3.203 (0.123)	<.001	−1.925 (0.094)		<.001	−2.259 (0.098)		<.001	−1.955 (0.167)		<.001	−1.136 (0.187)		<.001
Teeth lost	0.011 (0.112)	.92	−0.049 (0.081)		.54	0.342 (0.081)		<.001	−0.301 (0.158)		.06	−0.656 (0.228)		.004
Below poverty	−0.158 (0.064)	.01	−0.256 (0.044)		<.001	−0.328 (0.042)		<.001	−0.184 (0.088)		.04	−0.079 (0.113)		.48
Food stamps^a^	−0.281 (0.072)	<.001	−0.346 (0.047)		<.001	−0.156 (0.039)		<.001	−0.279 (0.103)		.007	−0.377 (0.121)		.002
Annual checkup	0.292 (0.110)	.008	0.520 (0.079)		<.001	1.022 (0.065)		<.001	−0.056 (0.162)		.73	0.563 (0.222)		.01
Lack of sleep	0.955 (0.158)	<.001	0.193 (0.108)		.07	0.048 (0.078)		.07	1.346 (0.232)		<.001	0.565 (0.305)		.01
Intercept	1.344 (0.044)	<.001	1.261 (0.025)		<.001	1.248 (0.037)		<.001	1.314 (0.034)		<.001	1.383 (0.042)		<.001

^a^Variables are statistically significant in all groups.

**Table 3:** City level–random effect results of the linear mixed-effect model.

	Model 1 (blue-low)	Model 2 (blue-mid)	Model 3 (blue-high)	Model 4 (red-low)	Model 5 (red-high)
City groups–intercept, variance (SD)	0.116 (0.340)	0.094 (0.307)	0.143 (0.378)	0.358 (0.189)	0.068 (0.260)
Residual, variance (SD)	0.050 (0.223)	0.064 (0.252)	0.055 (0.235)	0.054 (0.231)	0.059 (0.243)
Random effects	0.2225	0.1936	0.2861	0.0926	0.1416

**Table 4:** Overall model statistics and performance of the linear mixed-effect model.

	Model 1 (blue-low)	Model 2 (blue-mid)	Model 3 (blue-high)	Model 4 (red-low)	Model 5 (red-high)
Number of census tracts	3350	9803	9144	1991	1728
Number of city	73	198	120	53	55
*R*^2^ marginal—census tract level	0.6816	0.6759	0.6034	0.7690	0.7351
*R*^2^ conditional—city level	0.9041	0.8695	0.8895	0.8616	0.8767

## Discussion

### Principal Findings

In this study, we have demonstrated that, with the use of census tracts as the unit of analysis, more insights are extracted regarding how SDOH factors would affect the health outcomes of a diverse population. Furthermore, aggregating data into city jurisdictions enables a more targeted examination of how each city operates and influences health outcomes with an eye on policy generation and implementation at the city level.

Using a unique grouping method for cities based on population density and political affiliation, we reveal that cities are similar in many ways yet exhibit remarkable differences in public health. Specifically, we observed a major divide in public health and the different impacts of SDOH factors on population health outcomes between different political support in the United States, which contains only 2 main political parties. The results reinforce the current political climate in the United States, namely, the polarization between the 2 parties and how it affects health care outcome [69-71]. These findings add complexity for city officials who are monitoring and maintaining the quality of public health while upholding the political ideals and expectations through policy enforcement. Similarly, population density, which substitutes urban-rural designations, adds to the dichotomy of health. When cities are in the same grouping, they share many aspects of SDOH effects on health outcomes, although there is a distinct difference among members of different groups.

Our research yielded several interesting findings related to different SDOH factors. For example, we found that the number of adults who lost all their natural teeth before age 65 contributed to health outcomes in an oscillating manner. Although there is an established link between oral health and quality of life, there seems to be a disconnect between oral health and population health outcomes in the literature [72,73]. In addition, we observed the interrelationship between access to health services and annual checkups, highlighting the importance of health insurance for improving health outcomes. The health care system in the United States is complex, and obtaining health insurance for the disadvantaged group has been a major roadblock. Having health insurance is not enough; with it alone, there is only a negligible uptick in annual checkups [74]. These findings strongly support the need to establish incentives for having more individuals perform annual checkups. For example, the Preventive health Evidence-based Recommendation Form is a program that can potentially fulfill such a need [75].

This study has several implications for public health. First, we highlight that public health policies should differ among cities with different population densities and political affiliations. For instance, policies targeting the population with asthma, such as promoting environmental cleanliness, reducing pollution particles, and reducing the costs of asthma treatment and medication, would have a much stronger effect in red-leaning cities with a high population density than in cities in other groups. Likewise, smoking affects the blue-leaning cities the most, so a policy to discourage smoking and promote quitting would be more beneficial to population health outcomes in those cities. As resources are scarce, cities should allocate their resources according to the effectiveness of the proposed solutions. Therefore, each devised policy could target different SDOH factors for policy interventions to optimize health outcomes based on the categorization of each city. Second, our findings provide additional variables of interest for invigorating public health policy transfer possibilities among cities. Policy transfer is a well-studied phenomenon worldwide, especially in the European Union, but it has been much less studied in the United States. This study indicates that policy transfer between cities in the same group is possible. For example, a blue-leaning city with low population density might try to focus more on reducing smoking by perusing policies from health care offices residing in other blue-leaning rural cities. Similarly, cities may be able to pool resources together and procure a repository containing all related health policies. This repository could help facilitate faster and more efficient knowledge transfer between cities across the United States.

### Limitations

This study has several limitations. First, the curated data were not equivalent to those of the CHR. Only a handful of equivalent variables are available at the census tract level. Second, data normalization in the CHR encompasses all counties in the United States, although this study limits the data set to 499 cities, potentially skewing the results. Third, although the cities may represent many populations, sparse suburban areas and rural areas are largely neglected owing to data availability. Fourth, as an ecological study, there are possible confounders that existed outside our data set. Finally, the ecological nature of the study prohibits generalization of conclusions such as giving more food stamps to an individual, which could result in a change in personal health outcomes. Future research should continue to explore the relationship between SDOH and health outcomes in other delineations suitable for policy decision making.

### Conclusions

This study aimed to investigate the impact of SDOH factors on population health outcomes using a large data set comprising 29,126 census tracts within 499 cities across all 50 states in the United States. Our results identified 4 SDOH factors, namely, asthma, kidney disease, smoking, and food stamps, that have major effects across cities with different population densities and political affiliations. In addition, this study highlights the need for differentiated public health policies among cities with different population densities and political affiliations. The analysis of data at the city level, in which policies and decisions directly affect its citizens, promotes an understanding of how SDOH factors affect population health outcomes. The grouping mechanism, based on the combination of population density and political affiliation, provides a useful framework for separating and comparing different census tracts in different cities. To that end, this study adds to the existing literature on various ways to improve health equity among geographic areas or demographic and socioeconomic groups.

## Appendix

Multimedia Appendix 1 List of 499 cities, breaking down by 5 groups: blue-low, blue-mid, blue-high, red-low, and red-high.

Multimedia Appendix 2 List of the initial 148 independent variables.

## Abbreviations

CHR: County Health Ranking
SDOH: social determinants of health

## Data Availability

The data set will be available upon reasonable request to the corresponding author.
