# Supplementary material for: The Association Between Social Determinants of Health and Population Health Outcomes: Ecological Analysis
Source: JMIR Public Health Surveill. 2023 Mar 29;9:e44070. doi: 10.2196/44070 (PMC10131773; doi:10.2196/44070)
Supplement: Multimedia Appendix 1 [file publichealth_v9i1e44070_app1.docx]

Appendix 1

List of Cities Within Each Study Group

List of Blue-High cities

| Alameda, CA | Clearwater, FL | Houston, TX | Mountain View, CA | San Leandro, CA |
| --- | --- | --- | --- | --- |
| Alexandria, VA | Clifton, NJ | Huntington Beach, CA | New Haven, CT | San Marcos, CA |
| Alhambra, CA | Compton, CA | Inglewood, CA | New Rochelle, NY | San Mateo, CA |
| Allentown, PA | Concord, CA | Jersey City, NJ | New York, NY | Sandy, UT |
| Anaheim, CA | Coral Springs, FL | Kent, WA | Newark, NJ | Santa Ana, CA |
| Arlington Heights, IL | Corona, CA | Lakewood, CA | Norwalk, CA | Santa Barbara, CA |
| Baldwin Park, CA | Costa Mesa, CA | Largo, FL | Oakland, CA | Santa Clara, CA |
| Baltimore, MD | Daly City, CA | Lauderhill, FL | Oxnard, CA | Santa Monica, CA |
| Beaverton, OR | Downey, CA | Lawrence, MA | Palatine, IL | Schaumburg, IL |
| Bellflower, CA | El Cajon, CA | Long Beach, CA | Pasadena, CA | Schenectady, NY |
| Berkeley, CA | El Monte, CA | Los Angeles, CA | Passaic, NJ | Seattle, WA |
| Boston, MA | Elizabeth, NJ | Lowell, MA | Paterson, NJ | Somerville, MA |
| Boynton Beach, FL | Evanston, IL | Lynn, MA | Pawtucket, RI | South Gate, CA |
| Bridgeport, CT | Fontana, CA | Lynwood, CA | Philadelphia, PA | Sunnyvale, CA |
| Buena Park, CA | Fort Lauderdale, FL | Miami Beach, FL | Pomona, CA | Torrance, CA |
| Buffalo, NY | Fullerton, CA | Miami Gardens, FL | Providence, RI | Trenton, NJ |
| Burbank, CA | Garden Grove, CA | Miami, FL | Redondo Beach, CA | Tustin, CA |
| Cambridge, MA | Glendale, CA | Milpitas, CA | Rialto, CA | Union City, NJ |
| Camden, NJ | Gresham, OR | Milwaukee, WI | Rochester, NY | Vista, CA |
| Centennial, CO | Hartford, CT | Minneapolis, MN | Round Rock, TX | West Covina, CA |
| Chicago, IL | Hawthorne, CA | Mission Viejo, CA | Salinas, CA | Westminster, CA |
| Chula Vista, CA | Hialeah, FL | Missouri City, TX | San Bernardino, CA | Whittier, CA |
| Cicero, IL | Hillsboro, OR | Modesto, CA | San Francisco, CA | Wilmington, DE |
| Citrus Heights, CA | Hollywood, FL | Mount Vernon, NY | San Jose, CA | Yonkers, NY |

List of Blue-Mid cities

| Akron, OH | Dallas, TX | Irvine, CA | Omaha, NE | Scranton, PA |
| --- | --- | --- | --- | --- |
| Albany, NY | Davie, FL | Johns Creek, GA | Ontario, CA | Simi Valley, CA |
| Albuquerque, NM | Dayton, OH | Joliet, IL | Orange, CA | South Bend, IN |
| Ann Arbor, MI | Dearborn, MI | Kalamazoo, MI | Orlando, FL | Southfield, MI |
| Antioch, CA | Deerfield Beach, FL | Lafayette, IN | Overland Park, KS | Sparks, NV |
| Arlington, TX | Denver, CO | Lake Forest, CA | Parma, OH | Springfield, MA |
| Arvada, CO | Des Moines, IA | Lakewood, CO | Pasadena, TX | St. Louis, MO |
| Asheville, NC | Detroit, MI | Lansing, MI | Pembroke Pines, FL | St. Paul, MN |
| Atlanta, GA | Durham, NC | Laredo, TX | Peoria, IL | St. Petersburg, FL |
| Auburn, WA | Edinburg, TX | Las Vegas, NV | Perris, CA | Stamford, CT |
| Aurora, CO | El Paso, TX | Lawrence, KS | Pharr, TX | Stockton, CA |
| Aurora, IL | Elgin, IL | Lincoln, NE | Phoenix, AZ | Sugar Land, TX |
| Austin, TX | Elk Grove, CA | Livermore, CA | Pittsburgh, PA | Sunrise, FL |
| Baton Rouge, LA | Erie, PA | Longmont, CO | Plantation, FL | Syracuse, NY |
| Baytown, TX | Escondido, CA | Loveland, CO | Pleasanton, CA | Tacoma, WA |
| Bellevue, WA | Eugene, OR | Madison, WI | Pompano Beach, FL | Tampa, FL |
| Bellingham, WA | Everett, WA | Manchester, NH | Portland, ME | Temecula, CA |
| Bend, OR | Fairfield, CA | Manteca, CA | Portland, OR | Tempe, AZ |
| Bethlehem, PA | Federal Way, WA | McAllen, TX | Quincy, MA | Thornton, CO |
| Bloomington, IL | Flint, MI | Menifee, CA | Raleigh, NC | Thousand Oaks, CA |
| Bloomington, IN | Folsom, CA | Merced, CA | Rancho Cucamonga, CA | Toledo, OH |
| Boca Raton, FL | Fort Collins, CO | Mesa, AZ | Redlands, CA | Tracy, CA |
| Bolingbrook, IL | Fort Worth, TX | Mesquite, TX | Redwood City, CA | Tucson, AZ |
| Boulder, CO | Fremont, CA | Miramar, FL | Reno, NV | Turlock, CA |
| Brockton, MA | Fresno, CA | Mission, TX | Renton, WA | Union City, CA |
| Brooklyn Park, MN | Gainesville, FL | Missoula, MT | Richardson, TX | Upland, CA |
| Burlington, VT | Garland, TX | Moreno Valley, CA | Richmond, CA | Vacaville, CA |
| Carlsbad, CA | Gilbert, AZ | Murrieta, CA | Richmond, VA | Vallejo, CA |
| Carson, CA | Glendale, AZ | Napa, CA | Riverside, CA | Vancouver, WA |
| Cary, NC | Grand Prairie, TX | Naperville, IL | Rockford, IL | Waterbury, CT |
| Champaign, IL | Grand Rapids, MI | Nashua, NH | Sacramento, CA | Waukegan, IL |
| Chandler, AZ | Greensboro, NC | New Bedford, MA | Salem, OR | West Jordan, UT |
| Charlotte, NC | Greenville, NC | New Britain, CT | San Antonio, TX | West Palm Beach, FL |
| Chico, CA | Hammond, IN | Newport Beach, CA | Ventura, CA | West Valley City, UT |
| Chino, CA | Hayward, CA | Newton, MA | San Diego, CA | Westland, MI |
| Cincinnati, OH | Hemet, CA | Norfolk, VA | San Ramon, CA | Westminster, CO |
| Cleveland, OH | Henderson, NV | North Las Vegas, NV | Sandy Springs, GA | Worcester, MA |
| Clovis, CA | Hoover, AL | Norwalk, CT | Santa Clarita, CA | Wyoming, MI |
| Columbus, OH | Indio, CA | Oceanside, CA | Santa Maria, CA |  |
| Cranston, RI | Iowa City, IA | Olathe, KS | Santa Rosa, CA |  |

List of Blue-Low cities

| Albany, GA | Columbus, GA | Jacksonville, FL | Newport News, VA | Scottsdale, AZ |
| --- | --- | --- | --- | --- |
| Anchorage, AK | Danbury, CT | Kansas City, KS | North Charleston, SC | Shreveport, LA |
| Apple Valley, CA | Davenport, IA | Lancaster, CA | Palmdale, CA | Sioux City, IA |
| Athens, GA | Duluth, MN | Las Cruces, NM | Peoria, AZ | Suffolk, VA |
| Augusta, GA | Fall River, MA | Lexington, KY | Plymouth, MN | Surprise, AZ |
| Avondale, AZ | Farmington Hills, MI | Little Rock, AR | Portsmouth, VA | Tallahassee, FL |
| Birmingham, AL | Fayetteville, NC | Livonia, MI | Pueblo, CO | Topeka, KS |
| Bloomington, MN | Gary, IN | Louisville, KY | Rio Rancho, NM | Troy, MI |
| Brownsville, TX | Hampton, VA | Lynchburg, VA | Roanoke, VA | Victorville, CA |
| Cedar Rapids, IA | Hesperia, CA | Macon, GA | Rochester Hills, MI | Virginia Beach, VA |
| Charleston, SC | High Point, NC | Memphis, TN | Rochester, MN | Warwick, RI |
| Chesapeake, VA | Honolulu County, HI | Montgomery, AL | Roswell, GA | Waterloo, IA |
| Chino Hills, CA | Indianapolis, IN | Mount Pleasant, SC | Salt Lake City, UT | Winston-Salem, NC |
| Columbia, MO | Irving, TX | Nashville, TN | Santa Fe, NM |  |
| Columbia, SC | Jackson, MS | New Orleans, LA | Savannah, GA |  |

List of Red-High cities

| Allen, TX | Fishers, IN | Layton, UT | O'Fallon, MO | Spokane, WA |
| --- | --- | --- | --- | --- |
| Appleton, WI | Fort Wayne, IN | League City, TX | Ogden, UT | Springdale, AR |
| Bakersfield, CA | Frisco, TX | Lewisville, TX | Orem, UT | Springfield, MO |
| Billings, MT | Greeley, CO | McKinney, TX | Pearland, TX | Sterling Heights, MI |
| Boise City, ID | Green Bay, WI | Medford, OR | Plano, TX | Visalia, CA |
| Canton, OH | Kenner, LA | Melbourne, FL | Provo, UT | Warner Robins, GA |
| Carrollton, TX | Kennewick, WA | Meridian, ID | Racine, WI | Warren, MI |
| Cheyenne, WY | Kenosha, WI | Muncie, IN | Reading, PA | Waukesha, WI |
| Colorado Springs, CO | Killeen, TX | Murfreesboro, TN | Rock Hill, SC | Wichita, KS |
| Evansville, IN | Knoxville, TN | Nampa, ID | Roseville, CA | Wilmington, NC |
| Fargo, ND | Lafayette, LA | Odessa, TX | Spokane Valley, WA | Yakima, WA |

List of Red-Low cities

| Abilene, TX | Concord, NC | Independence, MO | Mobile, AL | St. George, UT |
| --- | --- | --- | --- | --- |
| Amarillo, TX | Corpus Christi, TX | Jacksonville, NC | Norman, OK | St. Joseph, MO |
| Beaumont, TX | Decatur, IL | Jonesboro, AR | Oklahoma City, OK | Tulsa, OK |
| Broken Arrow, OK | Deltona, FL | Kansas City, MO | Palm Bay, FL | Tuscaloosa, AL |
| Bryan, TX | Denton, TX | Lake Charles, LA | Palm Coast, FL | Tyler, TX |
| Cape Coral, FL | Edmond, OK | Lakeland, FL | Port St. Lucie, FL | Waco, TX |
| Carmel, IN | Fayetteville, AR | Lawton, OK | Rapid City, SD | Wichita Falls, TX |
| Charleston, WV | Fort Smith, AR | Lee's Summit, MO | Redding, CA | Youngstown, OH |
| Chattanooga, TN | Gastonia, NC | Longview, TX | San Angelo, TX | Yuma, AZ |
| Clarksville, TN | Gulfport, MS | Lubbock, TX | Sioux Falls, SD |  |
| College Station, TX | Huntsville, AL | Midland, TX | Springfield, IL |  |
