# Supplementary material for: The Association Between Social Determinants of Health and Population Health Outcomes: Ecological Analysis
Source: JMIR Public Health Surveill. 2023 Mar 29;9:e44070. doi: 10.2196/44070 (PMC10131773; doi:10.2196/44070)
Supplement: Multimedia Appendix 2 [file publichealth_v9i1e44070_app2.docx]

Appendix 2

List of Initial 148 independent variables

1. Access to health insurance
2. Arthritis
3. Bring Drinking
4. High Blood Pressure
5. Taking BP Medication
6. Cancer
7. Current Asthma
8. Coronary Heart Disease
9. Annual Checkup
10. Cholesterol Screening
11. Colorectal Cancer Screening
12. COPD Chronic obstructive pulmonary disease
13. Core preventive services for older men
14. Core preventive services for older women
15. Current Smoking
16. Dental Visit
17. Diabetes
18. High Cholesterol
19. Chronic Kidney Disease
20. Lack of Physical Activity
21. Mammography
22. Mental Health
23. OBESITY Cr
24. PAP Smear Test
25. Physical Health
26. Sleep less than 7 hours
27. Stroke
28. Teeth Lost
29. Children under 6 years
30. population 6 to 18 years
31. Population 19 to 25 years
32. Population 26 to 34 years
33. Population 35 to 44 years
34. Population 45 to 54 years
35. Population 55 to 64 years
36. Population 65 to 74 years
37. Population 75 years and older
38. Population with disability
39. Income to Property Ratio
40. population 26 years and over Less than high school graduate percent
41. population 26 years and over High school graduate
42. Owner occupied housing units with a mortgage Second mortgage only
43. Owner occupied housing units with a mortgage home equity only
44. Owner occupied housing units with a mortgage ratio of value to income Less than 2.0
45. Owner occupied housing units with a mortgage ratio of value to income 2.0 to 2.9
46. Owner occupied housing units with a mortgage ratio of value to income 3.0 to 3.9
47. Owner occupied housing units with a mortgage ratio of value to income 4.0 or more
48. Median housing costs dollars
49. Children under 6 years living with one parent
50. Income below 50 percent of poverty level
51. income below125 percent of poverty level
52. income below150 percent of poverty level
53. income below185 percent of poverty level
54. income below200 percent of poverty level
55. income below300 percent of poverty level
56. income below400 percent of poverty level
57. income below500 percent of poverty level
58. Population below poverty level
59. Percent population below poverty level
60. Percent under 18 below poverty level
61. Percent Under 5 years below poverty level
62. percent 5 to 17 years below poverty
63. Median monthly housing costs
64. Gini Index
65. pop 6 to 17 years
66. pop 6 to 17 years living with one parent
67. pop 6 to 17 years living with one parent Pct
68. Carpooled to work
69. Carpooled to work time Less than 10 minutes
70. Carpooled to work time 10 to 14 minutes
71. Carpooled to work time 15 to 19 minutes
72. Carpooled to work time 20 to 24 minutes
73. Carpooled to work time 25 to 29 minutes
74. Carpooled to work time 30 to 34 minutes
75. Carpooled to work time 35 to 44 minutes
76. Carpooled to work time 45 to 59 minutes
77. Carpooled to work time 60 or more minutes
78. Travel time to work less than 5 mins
79. Travel time to work less than 10
80. Travel time to work 10 to 14 minutes
81. Travel time to work 15 to 19 minutes
82. Travel time to work 20 to 24 minutes
83. Travel time to work 25 to 29 minutes
84. Travel time to work 30 to 34 minutes
85. Travel time to work 35 to 39 minutes
86. Travel time to work 40 to 44 minutes
87. Travel time to work 45 to 59 minutes
88. Travel time to work 60 to 89 minutes
89. Travel time to work90 or more minutes
90. Pop 6years older In labor force
91. Pop 6years older In labor force employed
92. Pop 6years older In labor force unemployed
93. Pop 6years older Not In labor force
94. Civilian labor force
95. Carpooled Commuting
96. Total household income dollars
97. Total household less than 10,000
98. Total household 10,000 to 14,999
99. Total household 15,000 to 24,999
100. Total household 25,000 to 34,999
101. Total household 35,000 to 49,999
102. Total household 50,000 to 74,999
103. Total household 75,000 to 99,999
104. Total household 75,000 to 99,999.1
105. Total household 100,000 to 149,999
106. Total household 150,000 to 199,999
107. Total household 200,000 or more
108. Median household income dollars
109. Mean household income dollars
110. Mean SS Income dollars
111. Per capita income dollars
112. Pop With health insurance coverage
113. Pop No health insurance coverage
114. Under 6 years Living with one parent
115. Under 6 years Living with one parent Percent
116. 6 to 17 years Living with one parent
117. 6 to 17 years Living with one parent percent
118. 17 years under living with one parent
119. household with cash public assistance or Food Stamps
120. household with cash public assistance or Food Stamps percent
121. household with NO cash public assistance or Food Stamps
122. household with NO cash public assistance or Food Stamps percent
123. Car, truck, or van 10 to 14 minutes to work percent
124. Car, truck, or van drive alone 20 to 24 minutes percent
125. median housing cost
126. Housing units
127. household average Plumbing and water heating
128. Household average Physician services
129. Hospital room and services
130. Household average Hospital room and services
131. Household average Cigarettes
132. Household average other tobacco products
133. Household average Smoking accessories
134. Household average Marijuana
135. Alcoholic beverages
136. Household average Alcoholic beverages
137. Food
138. Household average Food
139. Housing
140. Household average Housing
141. Household average Sports, recreation, and exercise equipment
142. Water and sewerage maintenance
143. Household average Water and sewerage maintenance
144. Tobacco products and smoking supplies
145. Household average Tobacco products and smoking supplies
146. Water and other public services
147. Household average Water and other public services
148. Household average Food at home
